# Position Checking-Based Sampling Approach Combined with Attraction Point Local Optimization for Safe Flight of UAVs

**DOI:** 10.3390/s24072157

**Published:** 2024-03-27

**Authors:** Hai Zhu, Baoquan Li, Ruiyang Tong, Haolin Yin, Canlin Zhu

**Affiliations:** 1 School of Control Science and Engineering, Tiangong University, Tianjin 300387, China; zhai08669827@163.com (H.Z.); tong166159@163.com (R.T.); haolinyinws@gmail.com (H.Y.); 2 School of Communications and Information Engineering, Nanjing University of Posts and Telecommunications, Nanjing 210003, China; canlzhu@163.com

**Keywords:** sampling-based planner, unmanned aerial vehicle, motion planning

## Abstract

Trading off the allocation of limited computational resources between front-end path generation and back-end trajectory optimization plays a key role in improving the efficiency of unmanned aerial vehicle (UAV) motion planning. In this paper, a sampling-based kinodynamic planning method that can reduce the computational cost as well as the risks of UAV flight is proposed. Firstly, an initial trajectory connecting the start and end points without considering obstacles is generated. Then, a spherical space is constructed around the topological vertices of the environment, based on the intersections of the trajectory with the obstacles. Next, some unnecessary sampling points, as well as node rewiring, are discarded by the designed position-checking strategy to minimize the computational cost and reduce the risks of UAV flight. Finally, in order to make the planning framework adaptable to complex scenarios, the strategies for selecting different attraction points according to the environment are designed, which further ensures the safe flight of the UAV while improving the success rate of the front-end trajectory. Simulations and real-world experiment comparisons are conducted on a vision-based platform to verify the performance of the proposed method.

## 1. Introduction

Nowadays, with advantages such as high mobility, flexibility, and easy operation, quadcopter UAVs are popularly used in a variety of complex and dangerous environments. Particularly in tunnel exploration and emergency rescue scenarios without global positioning system (GPS) signals, UAVs can only rely on their own cameras, radars, and other sensors to continue to perform tasks, so vision-based perception and navigation algorithms have been the focus of more and more researchers. The UAV system is mostly composed of the four essential modules: environment perception, state estimation, motion planning, and dynamic control [1,2]. A collision-free, smooth, and dynamically feasible trajectory guarantees flight safety, which is generated by a motion planning module in working scenarios. Motion planning is generally divided into two parts: front-end discrete path search and back-end continuous trajectory optimization, aiming at generating a reference trajectory that satisfies the above three basic conditions for the controller to track [3,4]. Since quadrotor UAVs with small fuselages have limited computational resources of on-board computers, a major research objective is how to fully utilize the limited resources to generate front-end paths and back-end trajectories. The trade-off between front-end and back-end computational resource allocation is an issue that needs to be considered in motion planning [5,6,7].

Consider transforming the problem of resource trade-offs into one of maximizing the use of information in the planning process. In other words, it can also be described as accelerating the trajectory optimization process by obtaining higher quality paths through using more parameter information in the front-end path generation process. Therefore, if the front-end path generation takes into account dynamics of UAVs, the back-end trajectory optimization will effectively decrease its burden. Zhou et al. propose a kinodynamic path search method to find initial trajectories that are safe, dynamically feasible and require the shortest time in a discrete control space [8]. A lightweight yet effective topology-guided kinodynamic planner for the fast flight of a quadrotor with limited onboard computational resources is proposed in [9]. Firstly, the topology of the environment is constructed through start and end points; next, the dynamics are sampled at the periphery of topological structure; and finally, the reference trajectory is obtained through local optimization and adjustment, which largely reduces the number of sampling points. In order to avoid the case that some narrow slits in the environment may provide more optimal solutions, Ye et al., in [10], propose a sampling-based kinodynamic planning method combined with local optimization, but this increases the risk of UAV collisions.

Through the above research work, it can be found that the use of bias sampling can be used to detect the position of some UAVs where collisions are possible, discarding some unnecessary sampling points, which not only ensures the flight safety of UAVs but also reduces the cost of path generation. Therefore, this paper considers the design of safer trajectory generation strategies that will make full use of the information of the environment topology. It then incorporates gradient-based trajectory optimization methods into the local optimization process, meaning that the trajectory replanning is formulated as a nonlinear optimization problem, with trade-offs between smoothing, safety, and dynamic feasibility, which can easily deform infeasible trajectory segments into feasible ones with very high efficiency and low memory requirements. In this way, it will largely reduce the computational burden of back-end trajectory optimization and improve the robustness in complex environments.

In this paper, which employs information from a topology-guided graph [9], a kinodynamic planning algorithm combining a position checking-based sampling method and attraction point local optimization is proposed, which both ensures the safe flight of UAVs and reduces the computational cost to a certain extent. The main contributions are as follows:

1. With the topology construction of the environment, the initial trajectory connecting the start and end points does not take obstacles into account. A designed spherical space is formed by taking the lengths of the two points where the trajectory enters and leaves the obstacle as diameters, and the centers of the two points as spheres. In the node expansion process, a position-checking method that directly discards the sampled points in the designed sphere space is proposed.
2. In order to exploit the already available information, a reduction of unnecessary calculations in the rewiring process is proposed by judging whether the midpoint of line segment connecting the two points is in the sphere or not; if it is, this means that this branch does not need to be calculated.
3. Different attraction point generation strategies are designed according to the environment in the local optimization process, and then the gradient-based trajectory optimization is used to keep the trajectory away from the surface of obstacles, to ensure flight safety.

In this paper, Section 2 introduces some related research work. Section 3 describes methods for generating front-end trajectories using information about the environment topology constructed. Section 4 designs attractor generation strategies for local trajectory optimization. Section 5 conducts simulations and real-world experiments on the proposed method and analyzes the results. Section 6 concludes this article.

## 2. Related Work

### 2.1. Sampling-Based Planning

**Figure 1:**
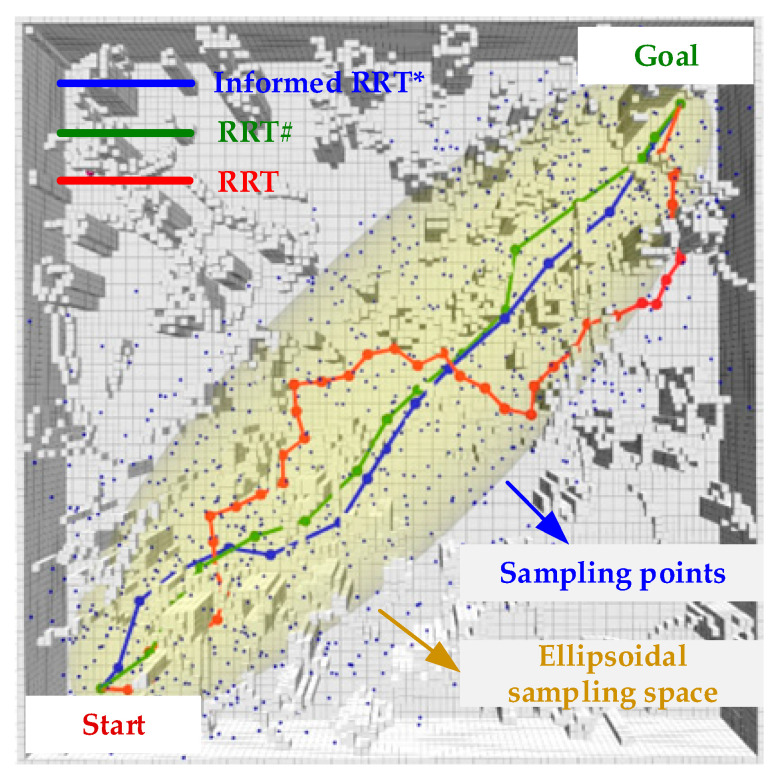
Diagram of the three sampling-based algorithms.

Search-based path generation schemes can find globally optimal solutions with sufficient computational resources and time. However, the magnitude of the space complexity of the algorithm grows exponentially with increasing dimensionality [11,12,13]. Sampling-based motion planning methods do not need to explicitly construct obstacle regions but instead use sampling to guide the search of configuration space (C-space). A large amount of C-space does not need to be explored and less on-board computational resources are consumed. Therefore, sampling-based algorithms are generally superior to search-based algorithms for mobile robots like UAVs that perform motion planning in high-dimensional spaces. The classical sampling-based algorithms are mainly divided into two categories: probabilistic roadmap (PRM) [14] based on global batch sampling and incremental iterative rapid exploration of random trees (RRT) [15]. Both types of algorithms choose the strategy for connecting the sampled nodes to the nearest nodes in the graph or tree, which is a fast way to explore the whole space, but does not utilize the existing structural information already available in the graph or tree. existing structural information in the graph or tree. As the demands of application scenarios increase, more and more algorithms based on them have been proposed. For example, the informed RRT* algorithm [16], which employs elliptical sampling instead of globally uniform sampling, is an incremental sampling-based method for optimal path planning. In addition, Arslan et al. in [17] proposed the RRT# algorithm, which does not rewire nonpromising nodes and uses cyclic rewiring to propagate the information obtained from sampling to the rest of the tree, improving the efficiency of the algorithm to converge on the optimal solution. In addition, Figure 1 gives a schematic diagram of the path search of the above three sampling algorithms in the same environment, with the same start and end points. As shown in Figure 1, compared to the other two algorithms, the RRT algorithm has the longest path in general because it does not have asymptotic optimality and samples randomly in the whole space. Both RRT# and Informed RRT* are based on the improvement of the RRT* algorithm with asymptotic optimality, and the path lengths are generally not much different, with the exception of being shorter, when compared to RRT. Informed RRT*will draw an ellipsoid space (gray-yellow area) with the start and end points as focuses after the end point is found; we will now sample this space (blue dots), so you can see that most of the blue points are in the ellipsoid space.

Boeuf et al. propose a quasi-metric to determine the proximity of a quadrotor and demonstrate that its integration into RRT-based and PRM-based algorithms reduces computational time by up to two orders of magnitude [18]. Karaman et al. show in [19] that the RRT* algorithm is an asymptotically optimal algorithm, which means that as time tends to infinity, the algorithm is able to find the optimal path. A sampling-based path search method, which allows sampling in regions where optimal solutions are more probable, is proposed in [20]. In addition, some sampling-based planning schemes incorporating neural networks are currently being proposed [21,22], which are effective in extracting the properties of high-dimensional features as a way of developing generalized methods to obtain probability distributions of samples, which do not only apply to specific scenarios and problems. However, it is difficult to obtain strong generalization ability with such methods, and the heavy network inference also makes it difficult to use these methods online in autonomous UAV navigation.

### 2.2. Kinodynamic Planning and Trajectory Optimization

The search-based kinodynamic planning scheme accomplishes path generation by using a discrete control space and searching for a segmented control solution through motion primitives. Liu et al. in [23] propose a search-based planning approach to find the smoothest and shortest time trajectories by exploring a map using a set of short time period motion primitives. Since sampling in state space, as in RRT*, tends to require solving the two-point boundary value problem (BVP) in optimal control, this means solving the equations of motion for a given dynamical system, given initial and termination conditions. This leads to inefficiency in expanding the tree in the high-dimensional state space, so this requires the design of a reasonable sampling strategy to minimize the unnecessary sampling points and accelerate the expansion of the tree, which will in turn improve the whole planning process.

Back-end trajectory optimization is essential to make the overall trajectory satisfy the dynamic demands of the whole system. The minimum snap trajectory generation algorithm proposed in [24] has been widely used in quadcopter UAV path planning efforts by solving a sequence of quadratic programming (QP) problems. Richter et al. in [25] prove that the minimum snap trajectories can be obtained by a closed-form solution, in which the trajectory is secured by iteratively adding intermediate track points. However, these general hard-constrained optimization schemes may cause a serious load on computational resources. Currently, the gradient-based soft constraint scheme is generally solved by modeling the trajectory optimization as a nonlinear optimization problem. In general, constructing a Euclidean signed distance field (ESDF) is necessary [26,27,28] to characterize constraints on safe obstacle avoidance, and provide information on the gradient of obstacle avoidance by linear interpolation in the ESDF, which has the advantage of being easy to compute but leads to the introduction of non-smooth functions. Of course, Zhou et al. propose a gradient-based planner without constructing an ESDF, which drastically reduces the amount of computation but also makes it easier for the UAV to fall into local optimality, and also degrades the trajectory quality [29]. In the motion planning process, trading off the resource allocation between the front and back ends also means minimizing the computational load of the on-board computer on the basis of ensuring the safety and feasibility of the reference trajectory.

## 3. Kinodynamic RRT* Planning Combined with Position Checking

### 3.1. Optimal State Transfer Cost

Based on the classic RRT* planning algorithm, the kinodynamic RRT* planning algorithm requires the calculation of state transfer cost between two nodes. Webb et al. in [30] construct a nilpotent dynamics matrix to find closed-form solutions for the two-point BVP, with the aim of saving computational cost. In addition, with the differential flatness property of a quadrotor UAV, state variables of the whole system can be represented by the four flat outputs (position vector $p(t)={\left[p_{x}(t),p_{y}(t),p_{z}(t)\right]}^{T}$, yaw φ(t)) and their derivatives. Thus, state transition can be modeled as a linear system:

(1) $$\dot{x}(t)=Ax(t)+Bu(t)$$

where system states and control input with jerk are shown as follows:

(2) $$\begin{array}{c} x(t)={\left[p(t)\;\dot{p}(t)\;\ddot{p}(t)\right]}^{T},\\ u(t)=\dddot{p}(t),\;\;\;\;\;\;\;\; \end{array}\;A=\left[\begin{array}{cc} 0_{6\times 3} & I_{6\times 6}\\ 0_{3\times 3} & 0_{3\times 6} \end{array}\right],\;B=\left[\begin{array}{c} 0_{6\times 3}\\ I_{3\times 3} \end{array}\right]$$

where $0_{r\times l}$ denotes the zero matrix for r rows and l columns, and $I_{e\times e}$ denotes the e-dimensional identity matrix.

Euclidean or Manhattan distance can be used for path planning if motion constraints are not considered. Optimal control is utilized for trajectory planning under motion constraints, and energy and time optimality are considered to design cost functions for state transition. If the transition cost between two states is small, they are affirmed to be close. Transition cost can be calculated based on arrival time τ and control input u(t), and the cost function from state $x_{0}$ to state $x_{1}$ is defined to be

(3) $$J(x_{0},x_{1})=\int_{0}^{\tau }\left(\rho +u{\left(t\right)}^{T}u(t)/2\right)dt$$

where ρ is a weight for time and control cost. For searching a trajectory from the initial state $x_{start}$ to the target state range space $X_{goal}$, the following model is established with computing optimal arrival time:

(4) $$\begin{array}{l} \underset{x(t)}{\min}J=\int_{0}^{\tau }\left(\rho +u{\left(t\right)}^{T}u(t)/2\right)dt\\ \;s.t.\;\;Ax(t)+Bu(t)-\dot{x}(t)=0,\\ \;\;\;\;x(0)=x_{start},x(\tau )\in X_{goal},\\ \;\;\;\;\forall t\in [0,\tau ],x(t)\in X^{free},u(t)\in U^{free} \end{array}$$

where $U^{free}$ denotes feasible control set, and $X_{goal}$ satisfies derivative constraints and does not contain obstacles. Solving an optimal trajectory edge between two neighboring state points in this paper is equivalent to solving the two-point BVP. During the node tree extension, the state at the start moment x(0) is fully given, while the state at the termination moment x(τ) is partially constrained. As for the node rewiring, the state at the starting moment x(0) and the state at the termination moment x(τ) are known nodes in the tree, so the constraints at both ends are fully given. In summary, the trajectory solution problem can be described as an optimal control problem with free terminal moments and constrained terminal states. The optimal arrival time and polynomial coefficients can be solved according to the Pontryagin’s maximum principle, so that the optimal trajectory $p^{*}(t)$ as well as the optimal control inputs $u^{*}(t)$ are obtained.

### 3.2. Kinodynamic RRT*

The kinodynamic RRT* algorithm is a sampling algorithm for solving path planning problems with dynamics constraints. It is an extension of the conventional RRT* algorithm that is intended to make it applicable to systems with continuous state and control input spaces, which is systematically proposed in [30], as shown in Algorithm 1.

**Figure 2:**
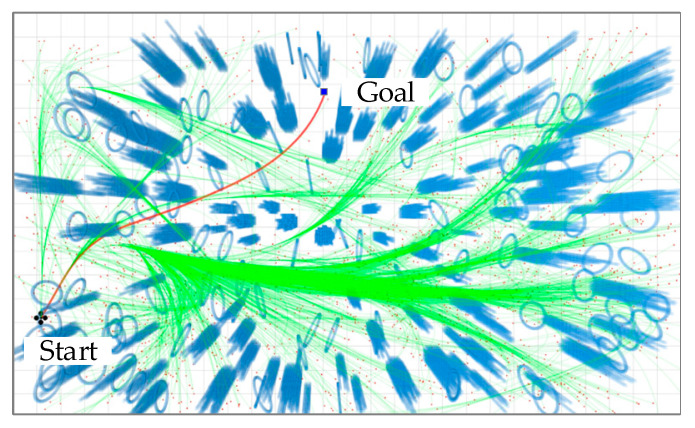
A snapshot of the demonstration of the kinodynamic RRT* algorithm.

Initially, the start point is used as the root node of the tree and an empty search tree T is initialized. Then a state $x_{new}$ is randomly sampled through RandomlySample $\left(x_{i},E\right)$, with position, velocity, and acceleration information obtained within a set number of samples and sampling time, which must satisfy dynamics constraints of the system. Next, FindNeighbor $\left(x_{new},T\right)$ and Expansion $\left(x_{new},X_{nearest}^{backward}\right)$ are used to find a state $x_{nearest}^{backward}$ around state $x_{new}$ that has the smallest transfer cost and serves as the parent node of $x_{new}$. Here, a check is made to see if the target region has been reached; if so, no further operations are needed and the searched tree T is returned. Instead, branches are rewritten using FinNeighbor $\left(x_{new},T\right)$ and E, which makes the resulting path asymptotically optimal. A gradual optimal path is gradually obtained through the above cycle of node tree extension as well as node rewiring. When the stopping condition of the loop is reached (e.g., the number of nodes in the trajectory tree reaches the set maximum value or a sampling time threshold is exceeded). Moreover, Figure 2 shows a demonstration case of the RRT* algorithm that considers the dynamics. As shown in Figure 2, the kinodynamic RRT* algorithm randomly samples in an environment with randomly distributed obstacles (light blue circles and bars). In this case, the green curve is the trajectory connecting the sampling points (red dots) and the start point; when the set goal (blue squares) is sampled, a red trajectory connecting the start and end points is generated, and the algorithm will terminate its operation.
**Algorithm 1:** Classical Kinodynamic RRT*1:**Notation:** Environment E, Tree T, Sampling State x, $x_{start}\in X^{free}$, $x_{goal}\in X^{free}$, Maximum Sampling Number n2:$T\leftarrow \{x_{start}\}$3:**for** i∈{1,n} **do**4:    $x_{new}\leftarrow$ RandomlySample $\left(x_{i},E\right)$;5:    $X_{nearest}^{backward}\leftarrow$ FindNeighbor $\left(x_{new},T\right)$;6:    **if**
$\left(X_{nearest}^{backward}\neq \emptyset \right)$
**then**7:        $\{x_{nearest}^{backward},x_{new}\}\leftarrow$ Expansion $\left(x_{new},X_{nearest}^{backward}\right)$; 8:        T← TreeUpdate $\left(\{x_{nearest}^{backward},x_{new}\},T\right)$;9:             **if** (getGoalRegional $\left(x_{new},x_{goal}\right)$) **then**10:                   **break**;11:             **end if**12:             $X_{nearest}^{forward}\leftarrow$ FindNeighbor $\left(x_{new},T\right)$;13:             **if**
$\left(X_{nearest}^{forward}\neq \emptyset \right)$
**then**14:                  Rewire $\left(X_{nearest}^{forward},T\right)$;15:             **end if**16:    **end if**
17:    **return**
T
18:**end for**

**Figure 3:**
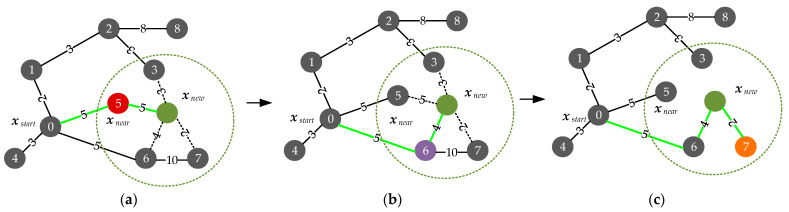

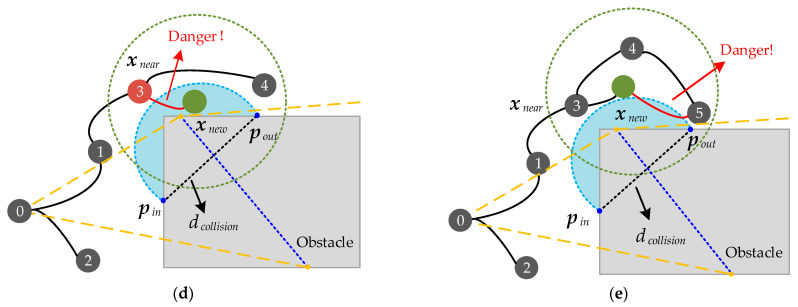
Diagram of the node expansion and node reconnection phases of the RRT* algorithm and motivation for node position checking in combination with the topology of the environment. (**a**) Find the lowest cost parent among neighbor nodes; (**b**) The new sampling point is reconnected to the lowest-cost parent node; (**c**) Use the new sampling point as a parent node to find less costly a path; (**d**) Motivation for adding sample point position checking to the node tree expansion process; (**e**) Motivation for adding sample point position checking to the node rewiring process.

In general, the two core steps of the RRT* algorithm, node tree extension and node rewiring are shown in Figure 3a–c below. In this case, the black circles indicate the nodes with numbers, Node 0 is the start node, and Node 5 is the nearest neighbor node $x_{near}$ of the new sampling point $x_{new}$; and the numbers on the path connecting the two nodes are the cost to be spent, which is generally obtained by solving the BVP if the UAV state is not considered. The space surrounded by the green circle drawn in dashed lines is the range of the parent node selected for the $x_{new}$. As shown in Figure 3a, since the cost of the path from Node 0 to $x_{new}$ via Node 6 is less than that of path to $x_{new}$ via any other node in the above range, Node 6 is set as the parent node of the new node, as shown by the green path in Figure 3b. In addition, as shown in Figure 3c, $x_{new}$ is set as the parent of the surrounding nodes, and compared to Node 6, the cost of the path of setting $x_{new}$ as the parent of Node 7 will become smaller, which means that the more optimal path is Node 0 to Node 7 via Node 6 and $x_{new}$, which also demonstrates the asymptotic optimality of the RRT* algorithm.

In this paper, through the above analysis of the two key steps of the RRT* algorithm, as shown by the orange dotted line in Figure 3d,e, the topology-guided graph [9] is constructed by using the two points $\left\{p_{in},p_{out}\right\}$ of initial trajectory through the obstacle and their perpendicular bisector (blue dashed line). The sampling guided by the guided graph of the environment will make the trajectory generated around $\left\{p_{in},p_{out}\right\}$ bring a high risk to the flight of the UAV. Therefore, in the node tree expansion as well as node rewiring stage, this paper exploits the information of the topology-guided graph to propose the strategy of dividing a spherical space (light blue area) with diameter size $d_{collision}$. It does this to check the position of sampling points, which not only reduces unnecessary BVP solving but also moves the trajectory far away from obstacles and ensures the safety of UAV flight. In particular, the spherical space construction method and the position-checking strategies are described in detail below.

### 3.3. Node Tree Extension Optimization

Considering that, as mentioned above, establishing a connecting trajectory between two states (namely, solving the two-point BVP between state nodes) consumes a large number of computational resources, the method of sampling the environment by roughly capturing the topology of the environment and guiding sampling around the periphery of the topology has been proposed. Sampling around the planar intersections of the topology of the environment can cause the problem of being too close to obstacles. Therefore, this paper considered utilizing the information of environment topology for position checking of the new sampling points, which not only reduces some ineffective sampling points but also ensures the safety of generated trajectories by expanding the sampling range.

**Figure 4:**
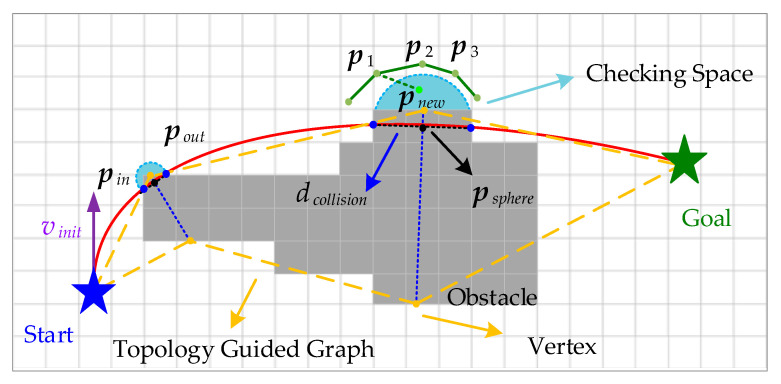
Expand tree nodes around vertices. Consider whether to add to the tree T by performing a position check on the new node.

Firstly, as shown in Figure 4, a red initial trajectory connecting the start and end points is generated by solving the two-point BVP without considering obstacles. Next, obstacle checking is performed to obtain two points $\left\{p_{in},p_{out}\right\}$ where the initial trajectory enters and leaves the obstacle. Then, the perpendicular bisector (blue dashed line) of the line connecting the two points is extended outward into the collision-free space and used as the vertices of the topology. In this way, a topology-guided graph that gives a rough indication of the environmental information is constructed. Finally, biased sampling is performed based on the constructed environment structure to improve the sampling efficiency.

In order to ensure that the sampling around the vertices can be as far away from the obstacles as possible, since $\left\{p_{in},p_{out}\right\}$ is closely related to them, it is considered to use the distance between two points to delimit a spherical checking space, whereby sampling points within this space cannot pass the position check. Specifically, position vector $p_{new}$ in the new sampling point $x_{new}$ is utilized to check:

(5) $$\begin{array}{l} d_{collision}={\left\|p_{out}-p_{in}\right\|}_{2},\\ p_{sphere}=(p_{out}-p_{in})/2,\\ \Delta d_{new}={\left\|p_{new}-p_{sphere}\right\|}_{2} \end{array}$$

where $d_{collision}$ is the diameter of the sphere, $p_{sphere}$ is the center of the sphere, and $\Delta d_{new}$ is the distance from the sampling point to the center of the sphere. If $\Delta d_{new}<d_{collision}/2$, which indicates that the sampling point is in the middle of the sphere space, this point is discarded.

This process is performed in Expansion $\left(x_{new},X_{nearest}^{backward}\right)$ in Algorithm 1, which ensures that other optimal or suboptimal sampling points are not affected and can be discarded before the two-point BVP is solved. In contrast to the algorithm in [10], a spherical space divided around the vertices of the topology-guided graph is proposed in the paper to perform a position check on the newly sampled points. This additional position checking not only reduces the number of triggers for local optimization of the back-end trajectory but also makes the flight safer.

### 3.4. Rewire Optimization

Unlike the RRT algorithm, the local optimality of the RRT* algorithm is safeguarded by the fact that it has a reconnection component that ensures its presence every time a new branch is successfully added to the node tree. This new sampling point is set to be the parent of a certain range of nodes in the surrounding area, and then the lower cost branch is selected by the cost change. However, the computational cost of the BVP is large in sampling-based planners, and thus some unnecessary solving needs to be avoided.

**Figure 5:**
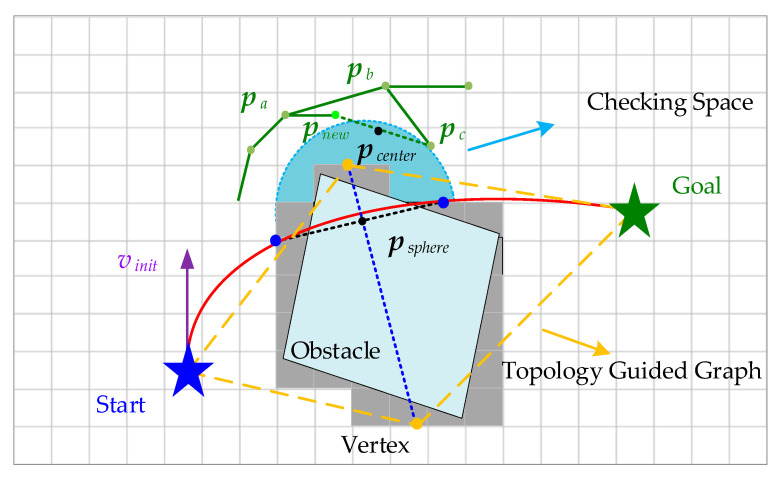
Considering the position-checked rewire to reduce the unnecessary two-point BVP solving process.

As described in Section 3.2, if motion constraints are not considered, the Euclidean distance or the Manhattan distance can be used. In contrast, in a state space with motion constraints, the introduction of optimal control is considered. Therefore, as shown in Figure 5, consideration can be given to make full use of the sphere with diameter $d_{collision}$, which is solved in the above section by assuming that nodes $p_{a},p_{b},p_{c}$ exist around the newly added node $x_{new}$ in the tree, and next treating $x_{new}$ as the parent of the surrounding nodes, before judging it by Rewire $\left(X_{nearest}^{forward},T\right)$ against $\{p_{new},p_{j}\},j=a,b,c$, before solving the BVP by setting the judging condition as

(6) $$\begin{array}{l} p_{center}=(p_{new}-p_{c})/2,\\ \Delta d_{center}={\left\|p_{sphere}-p_{center}\right\|}_{2} \end{array}$$

where $p_{center}$ is the midpoint between the parent node $p_{new}$ and child node $p_{c}$. Δd is the distance from the midpoint to the center of the sphere. If $\Delta d_{center}<d_{collision}/2,$ it shows that the reconnection of these two nodes is not necessary. Therefore, by checking the position of the midpoints of the above connecting lines in the rewiring phase, there will be less meaningless BVP solving, which also prevents the generation of high-risk reference trajectories. Algorithm 2 shows the implementation process of node tree expansion and reconnection.
**Algorithm 2:** Kinodynamic RRT* with Position Check1:**Notation:** Environment E, Tree T, Sampling State x, $x_{start}\in X^{free}$, $x_{goal}\in X^{free}$, Maximum Sampling Number n, $\left\{p_{in},p_{out}\right\}$2:$T\leftarrow \{x_{start}\}$3:**for** i∈{1,n} **do**4:    $x_{new}\leftarrow$ RandomlySample $\left(x_{i},Ε\right)$;5:    $X_{nearest}^{backward}\leftarrow$ FindNeighbor $\left(x_{new},T\right)$;6:    **if**
$\left(X_{nearest}^{backward}\neq \emptyset \right)$
**then**7:        **if**
$\left(\{p_{in},p_{out}\}\neq \emptyset \right)$ **then**8:             $d_{collision}\leftarrow$ ComputValue **()**;9:             $p_{sphere}\leftarrow$ ComputVector **()**;10:             $\Delta d_{new}\leftarrow$ ComputValue **()**;11:             **if**
$\left(\Delta d_{new}<d_{collision}/2\right)$ **then    **
12:                  **continue**;13:             **end if**14:        **end if    **        15:        $\left\{x_{nearest}^{backward},x_{new}\right\}$ ← Expansion $\left(x_{new},X_{nearest}^{backward}\right)$; 16:    **end if**17:    T← TreeUpdate $\left(\{x_{nearest}^{backward},x_{new}\},T\right)$;18:    **if** getGoalRegional $\left(x_{new},x_{goal}\right)$ **then**19:        **break**;20:    **end if**21:    $X_{nearest}^{forward}\leftarrow$ FindNeighbor $\left(x_{new},T\right)$;22:    **while** ($\left(X_{nearest}^{forward}\neq \emptyset \right)$) **do**23:        **if**
$\left(\{p_{in},p_{out}\}\neq \emptyset \right)$
**then**24:             $p_{center}\leftarrow$ ComputVector **()**;25:             $\Delta d_{center}\leftarrow$ ComputValue **()**;26:             **if**
$\left(\Delta d_{center}<d_{collision}/2\right)$ **then**27:             **Continue**;28:             **end if**29:        **end if**30:        Rewire $\left(X_{nearest}^{forward},T\right)$;31:    **end while**32:**end for**33:**return**T

## 4. Local Trajectory Optimization

In the above section, the segmented fifth degree polynomial trajectory has been obtained by solving the optimal control problem. It is easy to see from the above Figure 4 and Figure 5 that, since the constructed environment is very rough, relying only on this original trajectory for generating the final reference trajectory will result in a degradation of the quality of the generated trajectory. Therefore, a local optimization triggered by collision detection is proposed in [10] where, instead of direct trajectory discarding when a collision occurs in the generated trajectory, the failing trajectory is reused and locally adjusted by a gradient-based optimization scheme.

### 4.1. Optimization Problem Modeling

For systems with differential flatness property, each flat output dimension can be decoupled independently for planning. In particular, for a quadrotor UAV system, the state of the whole system can be represented by the four flat outputs (position: $p_{x},p_{y},p_{z}$, yaw angle: φ) and their derivatives as state variables, which are not considered in this paper with respect to their yaw angle φ.

A complex trajectory often needs to be represented by more than one polynomial in each dimension in space, and it is possible to divide the complete trajectory into multiple segments according to time t. Suppose the i-th segment of a trajectory $p_{i}(t)$ expressed by k segments, n-th degree polynomial is

(7) $$p_{i}(t)=c_{k,0}+c_{k,1}t+c_{k,2}t^{2}+\cdots +c_{k,n}t^{n}=c_{i}^{T}t,$$

where $c_{i}\in {\mathbb{R}}^{n+1}$ denotes the coefficients of the i-th trajectory, $t={\left(1,t,t^{2}\ldots ,t^{n}\right)}^{T}$, and $t\in [T_{i-1},T_{i}]$. The objective of the optimization is to find the optimal coefficients for each trajectory. In this paper, the coefficient $c_{i}$ of each segment of the trajectory is chosen to be solved to minimize the total cost of jerk and to satisfy the constraints, hence this is an optimization problem with constraints.

The triple integrator model, with jerk as the input, will constrain the position, velocity and acceleration of the head and tail of the trajectory p(t), and hence six equations are needed for constraints. Specifically, $p_{i}(t)$ has six coefficients when n=5, and then p(t) can be expressed completely by Equation (7), as

(8) $$p(t)=\left\{\begin{array}{l} c_{1,0}+c_{1,1}t+\cdots +c_{1,4}t^{4}+c_{1,5}t^{5},T_{0}\leq t\leq T_{1},\\ c_{2,0}+c_{2,1}t+\cdots +c_{2,4}t^{4}+c_{2,5}t^{5},T_{1}\leq t\leq T_{2},\\ \begin{array}{cc} \begin{array}{cc} \vdots & \end{array} & \end{array}\\ c_{k,0}+c_{k,1}t+\cdots +c_{k,4}t^{4}+c_{k,5}t^{5},T_{k-1}\leq t\leq T_{k} \end{array}\right.$$

where $t\in [T_{0},T_{k}]$ denotes the time period of the whole trajectory.

Therefore, the objective function Jerk(t) can be determined as

(9) $$Jerk(t)=p^{\left(3\right)}(t)=[0\;0\;0\;6\;24t\;60t^{2}]c,$$

where $c={\left[c_{1}^{T}\;c_{2}^{T}\cdots c_{k}^{T}\right]}^{T}={[c_{1,0}\cdots c_{1,5};c_{2,0}\cdots c_{2,5};\;\cdots \;]}^{T}$. In order to minimize it, meaning to minimize the integral of Jerk(t) over the whole time, it is usual to choose the 2-Norm for the solution, which can be converted to

(10) $$\underset{c}{\min}\;Jerk(t)=\underset{c}{\min}\sum_{i=1}^{k}\int_{T_{i-1}}^{T_{i}}{\left(p^{\left(3\right)}(t)\right)}^{2}dt.$$

By setting $a={\left[0\;0\;0\;6\;24t\;60t^{2}\right]}^{T}$, the transformed objective function is obtained

(11) $${\left(p^{\left(3\right)}(t)\right)}^{2}={\left(a^{T}c\right)}^{T}(ca)=c^{T}aa^{T}c.$$

Next, let $A=aa^{T}$, $\int_{T_{i-1}}^{T_{i}}{\left(p^{\left(3\right)}(t)\right)}^{2}dt$ in the objective function be converted to

(12) $$\int_{T_{i-1}}^{T_{i}}{\left(p^{\left(3\right)}(t)\right)}^{2}dt=\int_{T_{i-1}}^{T_{i}}c^{T}A(t)cdt=c^{T}\int_{T_{i-1}}^{T_{i}}A(t)dtc.$$

Calculating the integral part of this separately, a positive definite matrix $Q_{i}$ is obtained:

(13) $$Q_{i}=\int_{T_{i-1}}^{T_{i}}A(t)dt={\left[\begin{array}{cc} 0_{3\times 3} & 0_{3\times 3}\\ 0_{3\times 3} & \begin{array}{lll} 36t & 72t^{2} & 120t^{3}\\ 72t^{2} & 192t^{3} & 360t^{4}\\ 120t^{3} & 360t^{4} & 720t^{5} \end{array} \end{array}\right]}_{T_{i-1}}^{T_{i}}.$$

As a result, the optimization problem is converted into

(14) $$\begin{array}{l} Jerk(t)=\sum_{i=1}^{k}\int_{T_{i-1}}^{T_{i}}{\left(p^{\left(3\right)}(t)\right)}^{2}dt=\sum_{i=1}^{k}c_{i}^{T}Q_{i}c_{i}\\ =[c_{1}^{T}\;c_{2}^{T}\cdots c_{k}^{T}]\left[\begin{array}{cccc} Q_{1} & & & \\ & Q_{2} & & \\ & & \ddots & \\ & & & Q_{k} \end{array}\right]\left[\begin{array}{c} c_{1}\\ c_{2}\\ \vdots \\ c_{k} \end{array}\right]=c^{T}Qc. \end{array}$$

Since the objective function is a quadratic form, the trajectory optimization problem can be transformed into a QP problem. Where the objective function is of quadratic form and the matrix of the quadratic terms is positive definite, a closed-form solution is enabled to keep the trajectory away from obstacles by designing the objective term of the collision avoidance cost. A number of schemes have been proposed on this basis to make the resulting UAV trajectory converge on being smooth and collision free, and not exceeding the velocity constraints [31,32].

### 4.2. Local Optimization Strategy Adjustment

As shown in Figure 4 and Figure 5, the front-end trajectory tends to converge on the safe space based on discarding some sampling points and reducing the number of times of solving the BVP. However, in some complicated scenarios, such as very irregular obstacles, especially around $p_{in}$ and $p_{out}$, the state sampling points are close to obstacles, which results in the back-end optimized trajectory, bringing the risk of collision with obstacles to the UAV. In this paper, the quadratic objective function in Equation (14) consists of a smoothing cost term $J_{s}$, a trajectory position error cost term $J_{p}$, and a collision cost term $J_{c}$ in each dimensional space, and thus the optimization problem is modeled as

(15) $$\min J=\lambda_{s}J_{s}+\lambda_{p}J_{p}+\lambda_{c}J_{c},$$

where $\lambda_{s},\lambda_{p},\lambda_{c}$ are corresponding weight parameters.

In the local optimization, to avoid confusion with $\left\{p_{in},p_{out}\right\}$ above, for a collision trajectory edge, the collision start and collision end points are denoted respectively as $\left\{p_{start},p_{end}\right\}$. When $\left\{p_{start},p_{end}\right\}$ exists, the A* algorithm, a heuristic search algorithm for low-cost path, is utilized to search for a path $\Phi_{A*}$ from $p_{start}$ to $p_{end}$ on the surface of the obstacle, and a direction vector is used to guide the trajectory away from the obstacle by using the path points of $\Phi_{A*}$ and the center of the sphere $p_{sphere}^{\prime }$, similar to the one constructed in Section 3.3. The direction vector is used to guide the trajectory away from the obstacle. Due to the irregularity of the obstacles, it is not reasonable to consider only the midpoint $p_{m}$ of $\Phi_{A*}$, as this does not take into account the different levels of risk around points $p_{start}$ and $p_{end}$. Therefore, more refined attraction point $p_{att}$ generation strategies need to be designed.

**Figure 6:**
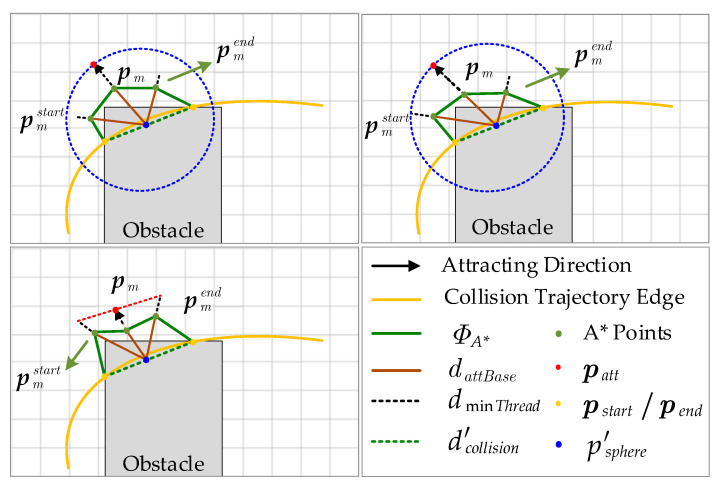
Diagram of the selection strategy of attraction points $p_{att}$ in the process of regional trajectory optimization.

Specifically, an intuitive demonstration is shown in Figure 6, where the path points of $\Phi_{A*}$ are divided into two parts, $\Phi_{A*}^{start}$ and $\Phi_{A*}^{end}$, by the midpoint $p_{m}$ of $\Phi_{A*}$, and then the midpoints $p_{m}^{start}$ and $p_{m}^{end}$ of the two parts are obtained, respectively. Next, the distance $d_{attBase},attbase\in (start,m,end)$ from these three points $p_{m},p_{m}^{start},p_{m}^{end}$ to the center of the sphere are computed, similarly to Equation (5). In the upper two cases in Figure 6, $p_{att}$ is obtained by

(16) $$p_{att}=\max(d_{attBase}+d_{\min Thread})$$

where $d_{\min Thread}$ is a set minimum value to prevent A* path points from being too close to obstacles and reducing the effectiveness of local optimization. Notably, the blue dashed line is used to visualize the maximum value and has no practical engineering significance.

In the third case, $p_{att}$ is determined according to the intersection of the red dashed line connecting the extension points of $d_{start},d_{end}$ with the extension line of $d_{m}$. In addition, similar to the method in Section 3.3, the position of the selected attraction points is checked against the spherical space; if ${\left\|p_{att}-p_{sphere}^{\prime }\right\|}_{2}<d_{collision}^{\prime }$, it means that no local optimization is needed, and the trajectory is directly resolved. Instead, the collision cost term is designed in a style similar to Equation (14):

(17) $$\begin{array}{cc} J_{c} & ={\sum_{C_{att}}\int_{t_{s,att}}^{t_{e,att}}\left[p(t)-\left(\max(d_{attBase})+d_{\min Thread}\right)\right]}^{2}dt\\ & =\sum_{C_{att}}\sum_{i\in attSeg}{\left(c_{i}-c_{i}^{att}\right)}^{T}\int_{t_{s,i,attP}}^{t_{e,i,attP}}tt^{T}dt(c_{i}-c_{i}^{att})\\ & =\sum_{C_{att}}{\left(c-c^{att}\right)}^{T}Q_{c,att}(c-c^{att}) \end{array}$$

where $C_{att}$ denotes the set of attractive points, and attSeg denotes the trajectory of the corresponding segment. Different attraction points are selected by the difference of environment, meaning that therefore the algorithm proposed in this paper is able to achieve better results in cluttered environments, as compared to [10]. In addition, Algorithm 3 gives the detailed process.
**Algorithm 3:** Attraction Points Selected by Environment1:**Notation:** Environment Ε, $p_{start}$, $p_{end}$, $p_{sphere}^{\prime }$, $p_{att}$, $d_{\min Thread}$2:**while**$\left(\{p_{start},p_{end}\}\neq \emptyset \right)$ **do**3:    $\Phi_{A*}\leftarrow$ Search $\left(p_{start},p_{end},Ε\right)$;4:    $p_{m}\leftarrow$ GetMidpoint $\left(p_{start},p_{end}\right)$;5:    $\{\Phi_{A*}^{start},\Phi_{A*}^{end}\}\leftarrow$ DividePath $\left(\Phi_{A*},p_{start},p_{m},p_{end}\right)$;6:    $(p_{m}^{start},p_{m}^{end})\leftarrow$ EachMidpoint $\left(p_{m},\{\Phi_{A*}^{start},\Phi_{A*}^{end}\}\right)$;7:    $d_{attBase}\leftarrow$${\left\|p_{attBase}-p_{sphere}^{\prime }\right\|}_{2}$;8:    $d_{attBase}^{\prime }\leftarrow$$d_{attBase}+d_{\min Thread}$;9:    **if**
$\left(d_{m}^{\prime }\geq d_{start}^{\prime }∥d_{m}^{\prime }\geq d_{end}^{\prime }\right)$ **then**10:        $p_{att}\leftarrow$ GetMax $\left(d_{m}^{\prime },d_{start}^{\prime },d_{end}^{\prime }\right)$;11:    **else then**
12:        $p_{att}\leftarrow$ GetIntersection $\left(d_{m}^{\prime },d_{start}^{\prime },d_{end}^{\prime }\right)$;13:        d←${\left\|p_{att}-p_{sphere}^{\prime }\right\|}_{2}$; 14:        **if**
$\left(d>d_{collision}^{\prime }\right)$ **then**         15:             **return**
$p_{att}$;16:        **else then**17:             **break;**18:        **end if**
19:    **end if**20:**end while**

## 5. Simulation and Experiment Results

### 5.1. Simulation

Simulation tests are run on a laptop with Intel i5-10200H processor, 8 GB RAM, 512 GB SSD and operating system Ubuntu 18.04 with melodic version of Robotics Operating System (ROS). In this case, the thresholds of velocity and acceleration are set to 4.0 m/s and 4.0 m/s^2^, respectively, and the weighting factor in Equation (3) is set to 100.

**Figure 7:**
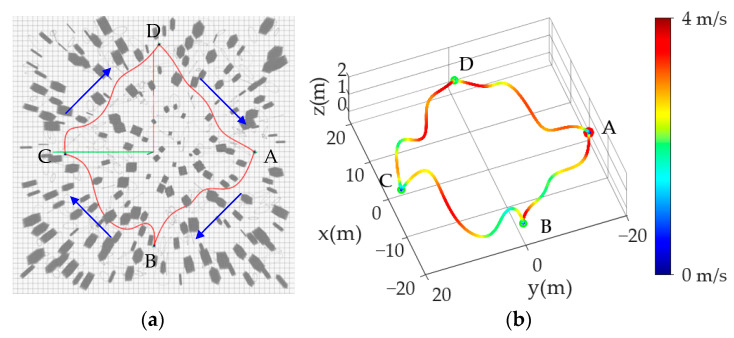
Demonstration of feasibility verification simulation. (**a**) Demonstration plot of randomly selected multiple targets (The red point A is both start and end point, and the green points B–D, selected according to the order of the blue arrows, are randomly selected intermediate target points); (**b**) UAV flight trajectories and the distribution of its velocity.

Simulation 1: Feasibility verification. The whole planning system is tested and verified in a cluttered environment with an area of 50 m × 50 m × 5 m and 400 obstacles. In this case, the obstacle color is set to gray to highlight the trajectory, instead of changing along the *Z*-axis direction, as in other tests. The start point is set as (0 m, −20 m, 1 m), and several points are randomly selected by 3D Nav Goal tool in Rviz for flight test; the flight process and the trajectory and the speed distribution are as shown in Figure 7. Through the simulation results, it can be seen that the UAV can complete the navigation task in the random scene to perform the flight task normally and completely.

**Figure 8:**
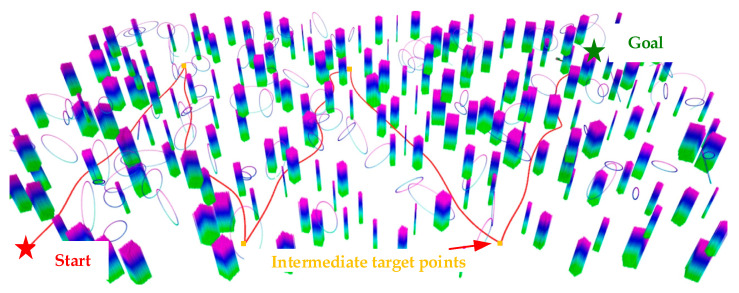
Feasibility validation in dense obstacle environments.

In order to make the results more general, a test is conducted in an environment with a size of 50 m × 100 m × 5 m, which contains 300 randomly distributed circular and cylindrical obstacles. As shown in Figure 8, the start point of the UAV (red pentagram) is set as (−22 m, 40 m, 1 m), and then the intermediate target points (orange squares) are randomly selected one by one through the 3DGoal tool in the interactive interface of the Rviz visualization, in which the green pentagram is the end point of the whole mission. It can be seen that the UAV does not collide with obstacles throughout the whole process, which indicates that the algorithms in this paper can successfully complete the mission.

**Figure 9:**
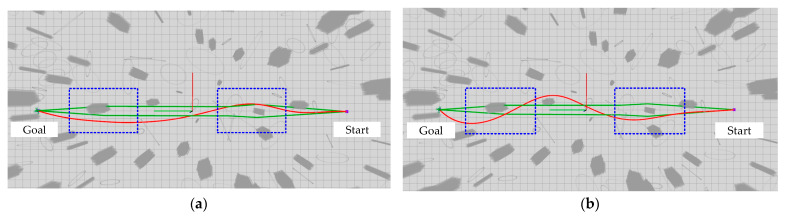
Comparative testing of trajectories around vertices of the guided graph of the environment. (**a**) Ref [10]; (**b**) Proposed.

Simulation 2: Trajectory comparative analysis. In order to verify the effectiveness of the method in this paper, the trajectories around the vertices of the guided graph of the environment are targeted for comparative analysis. Specifically, the size of the simulation environment is 50 m × 70 m × 5 m, the number of obstacles is 150, and the start point as well as the end point are set as (0 m, −20 m, 1 m) and (0 m, 20 m, 1 m), respectively. For the convenience of description, as shown in Figure 9, the trajectory color of both algorithms is uniformly set to red, the trajectories around the vertices are marked by blue dashed boxes, and the guided graph is indicated by green lines. Since the spherical space for position checking of the state points is delineated around the vertices, it can be seen in Figure 9 that the improved algorithm in this paper generates trajectories further away from the obstacles around the vertices, as compared to [10]. This indicates further that the algorithm proposed in this paper is able to reduce the risk of UAV flight around obstacles in order to ensure the safe flight of UAV.

**Figure 10:**
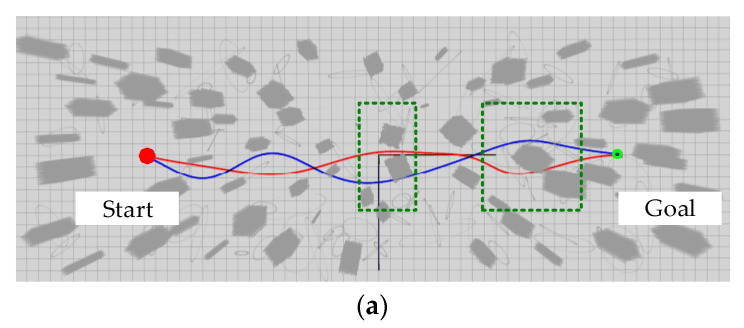

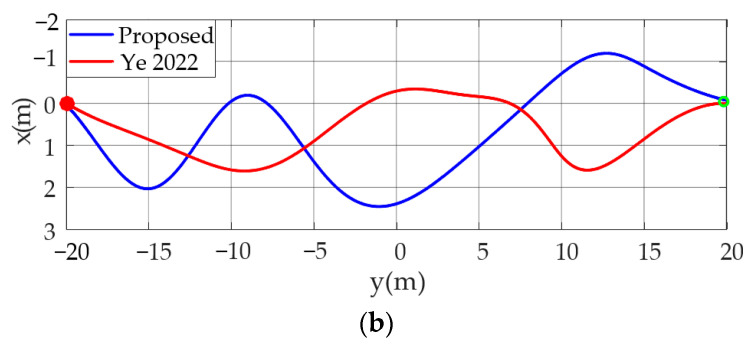
Comparison of the proposed method in this paper with the trajectory of [10]. (**a**) Rviz visualization results for both algorithms; (**b**) UAV trajectory in the X−Y plane.

**Figure 11:**
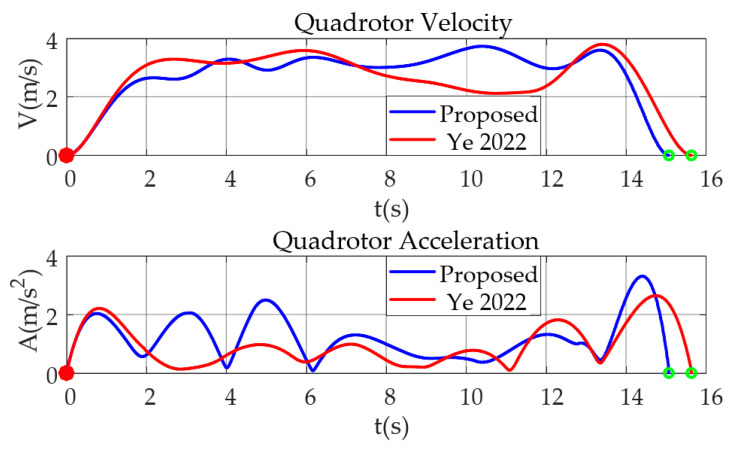
The proposed method in this paper compared with [10] for UAV velocity and acceleration comparison test. (The red dot is the start point and the green dot is the end point.).

In order to further verify the difference in the effectiveness of trajectories generated by kinodynamic RRT* algorithm with the addition of position checking in this paper, it is compared to the algorithm without the addition [10], in a comparison test conducted in an environment with a size of 60 m × 20 m × 5 m. Start and end points are fixed at (0 m, −20 m, 1 m) and (0 m, 20 m, 1 m), respectively. The number of obstacles is 200 and the resolution of the map is set as 0.1 m. Note from the results shown in Figure 10 that the length of the trajectory of the algorithm proposed in this paper is slightly longer than that of the comparison algorithm. However, as shown by the green dashed box in Figure 10a, the algorithm proposed in this paper clearly bypasses the narrowslit consisting of multiple obstacles. This also shows that, by increasing the position checking of the sampling points, the generated reference trajectory will avoid the more dangerous areas, thus reducing the risk of UAV collisions. In addition, Figure 11 gives the corresponding velocity and acceleration of the above trajectory, and the results further demonstrate the feasibility of the algorithm proposed in this paper.

**Figure 12:**
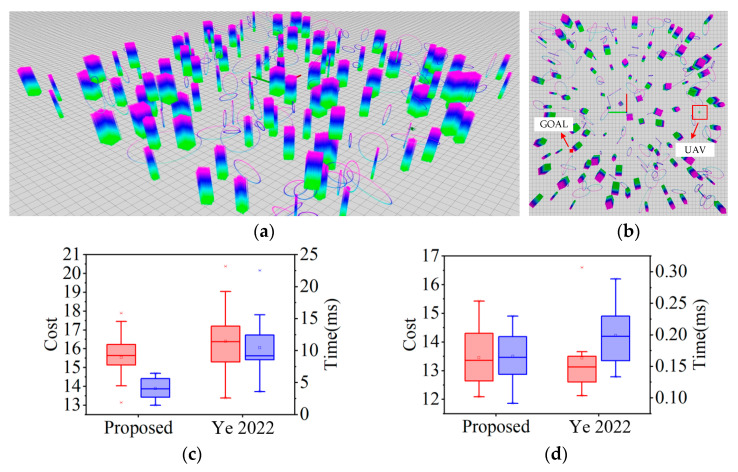
Performance comparison validation results. (**a**,**b**) Simulation environment; (**c**) Comparison of the distribution of front-end computational cost (red) and computational time (blue), with the highlighted horizontal line being the median; (**d**) Comparison of the distribution of back-end computational cost (red) and computational time (blue), with the highlighted horizontal line being the median [10].

Simulation 3: Performance analysis. The comparative simulation is validated in a cluttered environment with a map size of 50 m × 50 m × 5 m and a number of obstacles of 200. The start point is set as (0 m, −20 m, 1 m), the end point is set as (−10 m, −15 m, 1 m), and the resolution of the map is set as 0.1 m. Comparing 30 sets of comparative simulation experiments with [10], the computation time as well as the cost of the front-end and back-end trajectories are recorded, and the distributions are visualized in Figure 12a,b.

During the generation of the front-end trajectory, some points that may make the trajectory too close to the obstacle are discarded by position checking. Therefore, as shown in Figure 12c, the algorithm proposed in the paper has a smaller front-end solution time and computational cost compared with [10]. In addition, it is also shown in Figure 12c that the distribution of the results of the algorithm proposed in this paper is more centralized and the median is also smaller. Similarly, since the local optimization is also adopted in the front-end, the trajectory of the front-end already basically meets the flight requirements, so the trajectory optimization process of the back-end is triggered fewer times. As shown in Figure 12d, the computational cost of the algorithm in the paper is not much different from that of [10], but the computational time is reduced and the median computational time of the algorithm in the paper is lower.

### 5.2. Real-World Experiment

**Figure 13:**
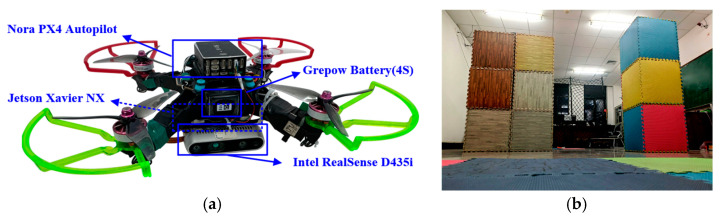
(**a**) Experimental platform; (**b**) Experimental scenario.

In order to test the effectiveness in executing a real flight mission, the algorithm proposed in this paper is implemented on the vision-based navigation UAV platform. As shown in Figure 13a, the experimental platform is constructed by referring to the Fast-Drone-250 project, with Intel RealSense D435i for perception and mapping in unknown environments. In addition, the onboard computer is Jetson Xavier NX (T503), with 8 GB RAM, 128 G of memory spaceand a pre-installed Ubuntu 18.04 operating system.

**Figure 14:**
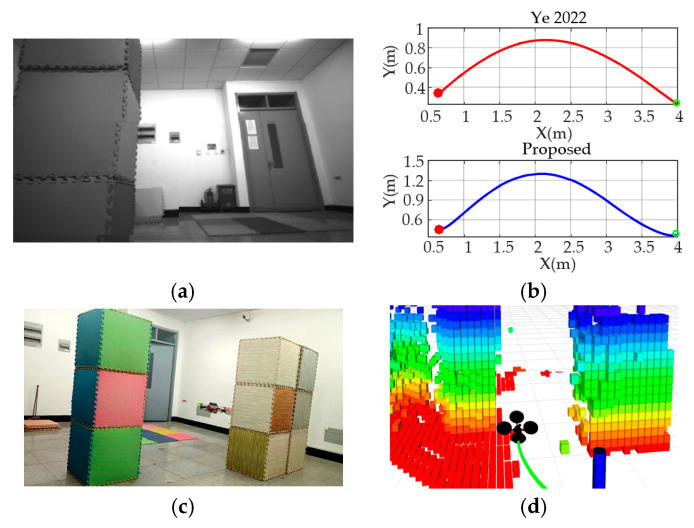
Comparing the test procedure as well as the demonstration results. (**a**) A snapshot of the UAV in flight; (**b**) The trajectories of two algorithms in the same test scenario (red: Ref [10], blue: the method proposed in this paper); (**c**) Image captured by the camera of the UAV in flight; (**d**) The display results in Rviz.

Experiment 1: Performance analysis. Experiments are conducted in a specific environment to validate the effectiveness of the algorithm proposed in this paper. Specifically, the end point is set as (4 m, 0 m, 1 m), and the thresholds of velocity and acceleration are set as 1.0 m/s and 0.8 m/s^2^, respectively. The work in this paper is based on the framework of [10] and so, in order to verify the effectiveness of the improved algorithm, two algorithms are executed separately, as depicted in the scenarios shown in Figure 13b. Figure 14a shows the grayscale image of the UAV viewpoint at a certain moment, which shows that the UAV is flying relatively stable and without shaking. Figure 14c,d show the captured picture of the third viewpoint at the same moment and the built-up picture in Rviz, respectively. The specific flight data results are displayed in Figure 14b, from which it can be seen that, in the process of avoiding the obstacles on the right side of the UAV, the algorithm in [10] deviates from the distance length of roughly 0.8~1 m, while the method in this paper deviates from the distance length of 1.2–1.5 m. Therefore, the results demonstrate the potential performance of the algorithm proposed in this paper to ensure the safe flight of UAVs.

**Figure 15:**
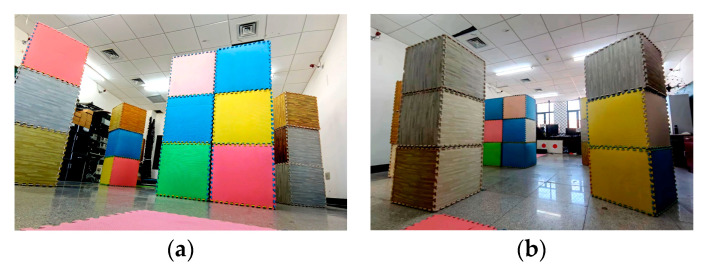
Randomly placed dense environments. (**a**) Start shooting views; (**b**) End shooting views.

**Figure 16:**
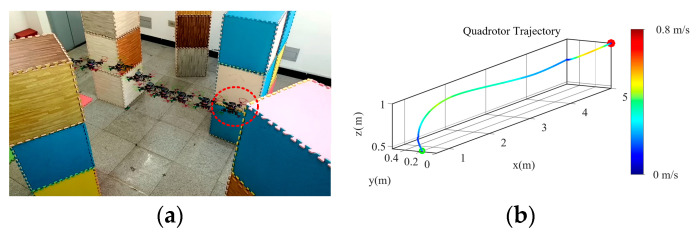

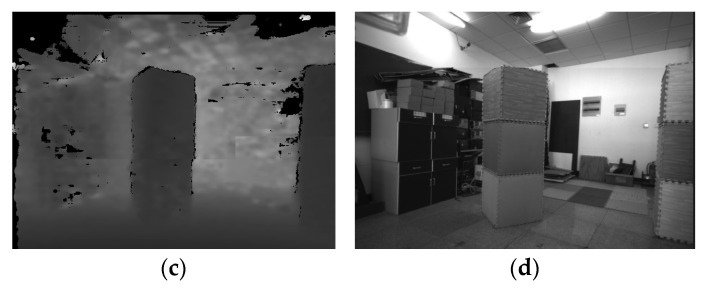
Experimental results in a randomized scenario. (**a**) Diagram of the UAV flight process; (**b**) The corresponding trajectory containing the velocity distribution; (**c**) Depth map of the UAV viewpoint circled with red in (**a**); (**d**) The corresponding grayscale map for the same viewpoint.

Experiment 2: Universality of the algorithm. In order to verify the effectiveness of the method proposed in this paper in a general scenario, a dense experimental environment with obstacles randomly placed in Figure 15 is constructed. The endpoint is set to (4.5 m, 0 m, 1 m), and the velocity and acceleration thresholds are set to 0.8 m/s and 1.0 m/s^2^, respectively. Figure 16a shows the process of the UAV in crossing the dense obstacle environment. Figure 16c,d are the binarized pictures of the UAV viewpoint in the red circle in Figure 16a, as well as the grayscale map, from which it can be seen that the UAV is flying more smoothly without violent shaking. The entire flight trajectory of the UAV and the velocity distribution on the trajectory are shown in Figure 16b, from which it can be seen that the velocity distribution is more uniform. Therefore, it also shows that the algorithm proposed for this problem has the potential to ensure the safety of UAV flight in a cluttered environment.

## 6. Conclusions

In this paper, by improving the tree extension and rewriting part of the classical kinodynamic RRT*, a position-checking strategy is proposed to be added to reduce the flight risk of UAVs as well as the unnecessary BVP solving. In addition, the reliability of UAVs in cluttered environments is also improved by local trajectory optimization based on attracting points. Firstly, the traditional RRT* algorithm is analyzed by introducing two core steps, namely, node tree expansion and node rewiring, as well as the existence of the problem that does not consider the body dynamics. Next, on the framework of the classical KRRT* algorithm, a position-checking strategy for new sampling points is proposed, which not only reduces the risk of trajectories around the vertices of the topology-guided graph but also reduces the unnecessary BVP solving. In order to improve the adaptability in cluttered environments, an algorithm for local trajectory optimization is proposed to select different attraction points according to the environment. Finally, it is verified through simulations and experiments that, compared to the existing work, the UAV motion planning algorithm proposed in this paper has a more secure trajectory, and the computational time and computational cost are reduced to some extent. Although the algorithm in this paper combines a local search-based algorithm, it is still a sampling-based algorithm overall and, due to the asymptotic optimality of the sampling algorithm, this leads to the occurrence of failing to find the optimal trajectory within a certain period of time. In the future, in order to improve the optimality of the trajectory, two aspects of research will be considered, namely, the selection of the trigger conditions of the search algorithm and the probability distribution of the sampling algorithm, so that the algorithm in this paper can be more effectively and efficiently applied in complex scenarios, enabling wider applications.

## Data Availability

The source code presented in this study is available on request from the corresponding author.
